# Benzopyranones from the Endophytic Fungus *Hyalodendriella* sp. Ponipodef12 and Their Bioactivities

**DOI:** 10.3390/molecules171011303

**Published:** 2012-09-25

**Authors:** Xiangjie Meng, Ziling Mao, Jingfeng Lou, Liang Xu, Lingyun Zhong, Youliang Peng, Ligang Zhou, Mingan Wang

**Affiliations:** 1 College of Agronomy and Biotechnology, China Agricultural University, Beijing 100193, China; 2 College of Science, China Agricultural University, Beijing 100193, China

**Keywords:** poplar hybrid ‘Neva’ endophytic fungus, *Hyalodendriella* sp. Ponipodef12, benzopyranones, palmariol B, 4-hydroxymellein, alternariol 9-methyl ether, botrallin, bioactivities

## Abstract

The endophytic fungus *Hyalodendriella* sp. Ponipodef12 was isolated from the hybrid ‘Neva’ of *Populus deltoides* Marsh × *P. nigra* L. In this study, four benzopyranones were isolated from the ethyl acetate extract of *Hyalodendriella* sp. Ponipodef12, and identified as palmariol B (**1**), 4-hydroxymellein (**2**), alternariol 9-methyl ether (**3**), and botrallin (**4**) by means of physicochemical and spectroscopic analysis. All the compounds were evaluated for their antibacterial, antifungal, antinematodal and acetylcholinesterase inhibitory activities. 4-Hydroxymellein (**2**) exhibited stronger antibacterial activity than the other compounds. Palmariol B (**1**) showed stronger antimicrobial, antinematodal and acetylcholinesterase inhibitory activities than alternariol 9-methyl ether (**3**) which indicated that the chlorine substitution at position 2 may contribute to its bioactivity. The results indicate the potential of this endophytic fungus as a source of bioactive benzopyranones.

## 1. Introduction

Endophytic fungi which inhabit normal tissues of host plants without causing apparent symptoms of pathogenesis are novel and rich sources of bioactive natural products which include alkaloids, amines, amides, steroids, terpenoids, isocoumarins, quinines, flavonoids, phenyl propanoids, lignans, phenols, aliphatics, *etc.* [1,2,3,4,5,6]. Due to this extensive potential, the objective of this work was to investigate the bioactive metabolites from an endophytic fungus *Hyalodendriella* sp. Ponipodef12 isolated from the healthy stems of the ‘Neva’ hybrid of *Populus deltoides* Marsh × *P. nigra* L. One benzopyranone that showed antimicrobial activity was previously obtained from this source and identified as botrallin [7]. In this work, three other benzopyranones were isolated for the first time from the endophytic fungus *Hyalodendriella* sp. Ponipodef12, identified, and their bioactivities, including antibacterial, antifungal, acetylcholinesterase inhibitory, and antinematodal properties, evaluated in order to provide data supporting the development and utilization of *Hyalodendriella* sp. Ponipodef12.

## 2. Results and Discussion

### 2.1. Elucidation of the Purified Benzopyranones

**Figure 1:**
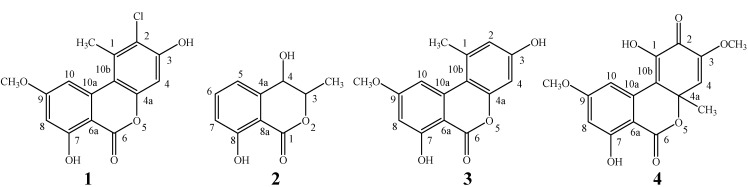
Chemical structures of the benzopyranones **1**–**4**.

Four compounds were isolated and purified from the crude ethyl acetate extract of the endophytic fungus *Hyalodendriella* sp. Ponipodef12. Their chemical structures were elucidated based on the physicochemical properties and spectral data, as well as by comparison of the properties and spectral characteristics with those in the literature. They were all known compounds, confirmed as 2-chloro-3,7-dihydroxy-9-methoxy-1-methyl-6*H*-dibenzo[*b*,*d*]pyran-6-one (=palmariol B, **1**) [8], 4,8-dihydroxy-3-methylbenzopyran-1-one (=4-hydroxymellein, **2**) [9], 3,7-dihydroxy-9-methoxy-1-methyl-6*H*-dibenzo[*b,d*]pyran-6-one (=alternariol 9-methyl ether, **3**) [10,11,12,13], and 1,7-dihydroxy-3,9-dimethoxy-4a-methyl-6*H*-dibenzo[*b,d*]pyran-2,6 (4a*H*)- dione (=botrallin, **4**) [14,15], respectively (Figure 1). Compounds **1**–**3** were isolated for the first time from the fungus *Hyalodendriella* sp. while the presence of compound **4** in this fungal strain was previously reported [7]. All these compounds belong to the class of benzopyranones with an isocoumarin skeleton.

Palmariol B (**1**) should derive from alternariol 9-methyl ether (**3**) by a chlorine substitution at position 2 (Figure 1). It was first isolated from the fungus *Lachnum palmae* derived from the decaying leaves of *Livistona* sp. [8]. This is the second time that palmariol B (**1**) was isolated from fungi. 4-Hydroxymellein (**2**) has been isolated from many fungi such as *Ascochyta* sp. [16], *Botryosphaeria mamane* [17], *Botryosphaeria obtusa* [9,18], *Botryosphaeria rhodina* [19], *Cercospora taiwanensis* [20], *Dinemasporium strigosum* [21], *Emericellopsis minima* [22], *Lasiodiplodia theobromae* [23], *Meliotus dentatus* [24], *Penicillium* sp. [25], *Seimatosporium* sp. [26], and *Septoria nodorum* [27]. Alternariol 9-methyl ether (**3**) has been isolated from some fungi such as *Alternaria brassicicola* [12], and *Lachnum palmae* [8]. Botrallin (**4**) was firstly isolated from the fungus *Botrytis allii* [14], and later it was isolated from the endophytic fungus *Microsphaeropsis olivacea* associated with a Chilean cupressaceous plant *Pilgerodendron uviferum* [15].

### 2.2. Antimicrobial Activity

Antimicrobial activities of the compounds with their median inhibitory concentration (IC_50_) values are shown in Table 1. The IC_50_ values of the compounds on *Agrobacterium tumefaciens* ranged from 18.22 μg/mL to 87.52 μg/mL. Those on *Bacillus subtilis*, *Pseudomonas lachrymans*, *Ralstonia solanacearum*, *Xanthomonas vesicatoria*, *Magnaporthe oryzae* ranged from 19.22 μg/mL to 98.47 μg/mL, 16.18 μg/mL to 92.21 μg/mL, 16.24 μg/mL to 85.46 μg/mL, 17.81 μg/mL to 86.32 μg/mL, 107.19 μg/mL to 123.19 μg/mL, respectively. On each microorganism, palmariol B (**1**) showed stronger antimicrobial activity than alternariol 9-methyl ether (**3**), which suggested that the chlorine substitution at position 2 may contribute to its antimicrobial activity. 4-Hydroxymellein (**2**) exhibited stronger antibacterial activity than the other compounds (*i.e*., **1**, **3** and **4**). Botrallin (**4**) exhibited relatively weaker antibacterial and stronger antifungal activity than the other compounds. Furthermore, the antibacterial activity results of the compounds were better than those for their antifungal activity. Taken together the results indicate the potential of the endophytic fungus *Hyalodendriella* sp. Ponipodef12 as a source of antimicrobial agents.

**Table 1:** Antimicrobial activity (IC_50_) of the benzopyranones.

Compd.	IC_50_ (μg/mL)					
	*A. tum.*	*B. sub.*	*P. lach.*	*R. sol.*	*X. ves.*	*M. or.*
Palmariol B (**1**)	19.35	27.67	16.74	17.51	28.11	118.72
4-Hydroxymellein (**2**)	18.22	19.22	16.18	16.24	17.81	113.40
Alternariol 9-methyl ether (**3**)	28.83	34.29	27.08	29.21	30.06	123.19
Botrallin (**4**)	87.52	98.47	92.21	85.46	86.32	107.19
CK^+^	6.34	8.35	8.34	7.19	6.79	13.01

Note: IC_50_, median inhibitory concentration; *A. tum.* = *Agrobacterium tumefaciens*; *B. sub.* = *Bacillus subtilis*; *P. lach.* = *Pseudomonas lachrymans*; *R. sol.* = *Ralstonia solanacearum*; *X. ves.* = *Xanthomonas vesicatoria*; *M. or.* = *Magnaporthe oryzae*; Positive controls for bacteria and fungus (*M. oryzae*) were streptomycin sulfate and carbendazim, respectively.

4-Hydroxymellein (**2**) from the endophytic fungus *Penicillium* sp. was reported to exhibit antifungal activity on *Cladosporium cladosporioides* and *C. sphaerospermum* at 5.00 μg/mL and 10.0 μg/mL, respectively [25] as well as antibacterial activity on *Staphylococcus aureus* and methicillin-resistant *S. aureus* with the equal MIC values of 8 μg/mL [17]. 4-Hydroxymellein (**2**) from the endophytic fungus *Meliotus dentatus* also showed strong antibacterial activity on *Bacillus megaterium* and antifungal activity on *Microbotryum violacerum* [24]. Alternariol 9-methyl ether (**3**) is a major mycotoxin produced by fungi of the genus *Alternaria* [28]. It can induce mitochondrial apoptosis in human colon carcinoma cells [29], and induce DNA strand breaks, micronuclei and gene mutations in various cultured mammalian cells [30]. Botrallin (**4**) was reported to show antifungal activity on the fungus *Alternaria alternata* with its MIC value as 62.5 μg/mL [15].

### 2.3. Antinematodal and Acetylcholinesterase Inhibitory Activities

Antinematodal activity on the nematode *Caenorhabditis elegans* and acetylcholinesterase inhibitory activity of the benzopyranones obtained by using the microplate assay expressed as the median inhibitory concentration (IC_50_) values are given in Table 2. The IC_50_ values of the antinematodal activity of the compounds ranged from 56.21 μg/mL to 93.99 μg/mL, and those for the acetyl-cholinesterase inhibitory activity ranged from 103.70 μg/mL to 135.52 μg/mL. Palmariol B showed stronger antinematodal and acetylcholinesterase inhibitory activities than alternariol 9-methyl ether. Botrallin (**4**) was previously reported to have acetycholinesterase (AChE) inhibitory activity with the IC_50_ value as 6.1 μg/mL [15]. The results indicate that the benzopyranones from the endophyte *Hyalodendriella* sp. Ponipodef12 have potential as antinematodal and insecticidal agents.

**Table 2:** Antinematodal activity on the nematode *Caenorhabditis elegans* and acetylcholinesterase inhibitory activity of the benzopyranones.

Compd.	IC_50_ (μg/mL)	
	Antinematodal activity on *C. elegans*	Acetylcholinesterase inhibitory activity
Palmariol B ( **1**)	56.21	115.31
4-Hydroxymellein ( **2**)	86.86	116.05
Alternariol 9-methyl ether ( **3**)	93.99	135.52
Botrallin ( **4**)	84.51	103.70
CK^+^	3.70	7.41

Note: IC_50_, median inhibitory concentration; Positive controls (CK^+^) for antinematodal and acetylcholinesterase inhibitory activities were avermectin and 9-amino-1,2,3,4-tetrahydroacridine hydrochloride hydrate, respectively.

## 3. Experimental

### 3.1. General

Melting points of the compounds were measured on an XT4-100B microscopic melting-point apparatus (Tianjin Tianguang Optical Instruments Company, Tianjin, China) and are uncorrected. NMR spectra were recorded on a Bruker Avance DRX-400 spectrometer (^1^H at 400 MHz and ^13^C at 100 MHz) using tetramethylsilane (TMS) as the internal standard, and chemical shifts were recorded as *δ* values. ESI-MS spectra were recorded on a Bruker Esquire 6000 LC/MS spectrometer and a Bruker Apex IV FTMS spectrometer. Both silica gel (200–300 mesh) for column chromatography (CC) and silica gel GF_254_ (10–20 mm) for thin layer chromatography (TLC) were acquired from the Qingdao Marine Chemical Company (Qingdao, China). Sephadex LH-20 was purchased from Pharmacia Biotech (Stockholm, Sweden). A microplate spectrophotometer (PowerWave HT, BioTek Instruments, Winooski, VT, USA) was employed to measure the light absorption value. Carbendazim, streptomycin sulfate, 9-amino-1,2,3,4-tetrahydroacridine hydrochloride hydrate, acetylthiocholine iodide (ATCI), 5,5'-dithio bis-(2-nitrobenzoic acid) (DTNB), and acetylcholinesterase (AChE) were purchased from Sigma-Aldrich (St. Louis, MO, USA). 3-(4,5-Dimethylthiazol-2-yl)-2,5-dephenyl tetrazolium bromide (MTT) was purchased from Amresco (Solon, OH, USA). All other chemicals and reagents were of analytical grade.

### 3.2. Endophytic Fungus and Fermentation

The endophytic fungus *Hyalodendriella* sp. Ponipodef12 (GenBank accession number HQ731647) was isolated from the healthy stems of the ‘Neva’ hybrid of *Populus deltoides* Marsh × *P. nigra* L. in our previous study [31]. It was stored both on PDA slants at 4 °C and in 40% glycerol at −70 °C in the Herbarium of the College of Agronomy and Biotechnology, China Agricultural University (Beijing, China). The fungus was cultured on PDA (potato 200 g/L, dextrose 20 g/L and agar 20 g/L) medium in Petri dishes at 25 °C for 10 days. For seed culture, two to three plugs of agar medium (0.5 × 0.5 cm) with fungal cultures were inoculated in each 250-mL Erlenmeyer flask containing 100 mL potato dextrose broth (PDB) medium, and incubated on a rotary shaker at 150 rpm and 25 °C for 5 days. For fermentation culture, about 50 mycelium pellets were inoculated in each 1,000-mL Erlenmeyer flask containing 300 mL PDB medium, and incubated on a rotary shaker at 150 rpm and 25 °C for 20 days. Afterwards, a total of 90 L fermentation broth was harvested. 

### 3.3. Extraction and Fractionation of the Compounds

The mycelia separated from the culture filtrate by filtration was lyophilized and ground to obtain 800 g of mycelia powder. This material was extracted with acetone (5 × 2 L). After filtration, the filtrate was concentrated under vacuum at 50 °C, the brown residue was suspended in water (1 L) and fractioned successively with petroleum ether (3 × 2 L), ethyl acetate (3 × 2 L) and *n*-butanol (3 × 2 L), to give their corresponding fractions. Meanwhile, the culture filtrate was concentrated to 1 L and fractionated with petroleum ether (3 × 2 L), ethyl acetate (3 × 2 L) and *n*-butanol (3 × 2 L), successively to obtain the respective fraction. As the results of TLC and TLC-bioautographic-assays were similar to each other for the ethyl acetate fractions from the mycelia and culture filtrate, both the ethyl acetate fractions were combined, and a total of 37.8 g concentrated crude ethyl acetate fraction was thus obtained.

The crude ethyl acetate fraction was firstly subjected to column chromatography (CC) over silica gel (200–300 mesh) eluted with CH_2_Cl_2_/MeOH (15:1, v/v) to obtain eight fractions (FA, FB, FC, FD, FE, FF, FG and FH) as monitored by TLC. Fraction FD was screened to show strong antimicrobial activity, and it was selected for further separation of the compounds on a silica gel column eluted with petroleum ether/CH_2_Cl_2_/MeOH (from 1:0:0 to 0:0:1, v/v) to give four subfractions. Subfraction FD-1 was purified by recrystalization to afford **1** (18 mg). FD-2 was purified by Sephadex LH-20 (CHCl_3_/MeOH = 1:1, v/v) and recrystalization to afford **2** (12 mg). FD-3 was further fractionated on a silica gel column eluted with CH_2_Cl_2_/MeOH (from 1:0 to 0:1, v/v), and purified by Sephadex LH-20 (CHCl_3_/MeOH = 1:1, v/v) and recrystalization to afford **3** (28 mg). FD-4 was purified by Sephadex LH-20 (CHCl_3_/MeOH = 1:1, v/v) and recrystalization to afford **4** (43 mg).

### 3.4. Physicochemical and Spectroscopic Data of the Benzopyranones

Compound **1** was isolated as a white amorphous powder (MeOH); m.p. 270–276 °C; UV (MeOH), λ_max_ 218, 257 nm; ESI-MS *m/z* 304.8 [M−H]^−^; The molecular formula C_15_H_11_ClO_5_ was assigned by HR-ESI-MS *m/z* 344.9927 [M+K]^+^ (calcd. 344.9932); ^1^H-NMR (DMSO-*d_6_*) *δ* (ppm): 2.79 (3H, s, CH_3_-1), 11.14 (1H, s, OH-3), 6.83 (1H, s, H-4), 11.64 (1H, s, OH-7), 6.61 (1H, d, *J* = 1.8 Hz, H-8), 3.90 (3H, s, OCH_3_-9), 7.15 (1H, d, *J* = 1.8 Hz, H-10); ^13^C-NMR (DMSO-*d_6_*) *δ* (ppm): 135.48 (C-1), 20.9 (CH_3_-1), 119.86 (C-2), 154.65 (C-3), 101.88 (C-4), 150.11 (C-4a), 163.96 (C-6), 98.59 (C-6a), 164.19 (C-7), 98.89 (C-8), 166.01 (C-9), 55.93 (OCH_3_-9), 104.50 (C-10), 136.87 (C-10a), 110.31 (C-10b). Those data were consistent with literature [8]. Therefore, compound **1** was identified as 2-chloro-3,7-dihydroxy-9-methoxy-1-methyl-6*H*-dibenzo [*b*,*d*] pyran-6-one, named palmariol B.

Compound **2 **was isolated as white dendrite-like crystals (MeOH); m.p. 110–114 °C; UV (MeOH), λ_max_ 246, 314 nm; The molecular formula C_10_H_10_O_4_ was assigned by HR-ESI-MS *m/z* 193.0507 [M−H]^−^ (calcd. 193.0501); ^1^H-NMR (CD_3_OD) *δ* (ppm): 4.56 (1H, m, H-3), 1.47 (3H, d, *J* = 6.0 Hz, CH_3_-3), 4.54 (1H, d, *J* = 3.6 Hz, H-4), 7.08 (1H, d, *J* = 7.4 Hz, H-5), 7.57 (1H, t, *J* = 8.4, 7.4 Hz, H-6), 6.93 (1H, d, *J* = 8.4 Hz, H-7), ^13^C-NMR (CD_3_OD) *δ* (ppm): 170.19 (C-1), 81.61 (C-3), 18.17 (CH_3_-3), 69.52 (C-4), 144.13 (C-4a), 117.71 (C-5), 137.80 (C-6), 117.80 (C-7), 162.89 (C-8), 108.01 (C-8a). Those data were consistent with literature [9,20]. Thus, compound **2** was identified as 4,8-dihydroxy-3-methylbenzopyran-1-one, named 4-hydroxymellein.

Compound **3** was isolated as a yellow amorphous powder (MeOH); m.p. 265–269 °C; UV (MeOH), λ_max_ 204, 256 nm; The molecular formula C_15_H_12_O_5_ was assigned by HR-ESI-MS *m/z* 273.0755 [M+H]^+^ (calcd. 273.0763); ^1^H-NMR (DMSO-*d_6_*) *δ* (ppm): 2.73 (3H, s, CH_3_-1), 6.72 (1H, d, *J* = 2.6 Hz, H-2), 11.34 (1H, s, OH-3), 6.64 (1H, d, *J* = 2.6 Hz, H-4), 11.82 (1H, s, OH-7), 6.61(1H, d, *J* = 2.5 Hz, H-8), 3.90 (3H, s, OCH_3_-9), 7.21 (1H, d, *J* = 2.6 Hz, H-10); ^13^C-NMR (DMSO-*d_6_*) *δ* (ppm): 138.45 (C-1), 25.03 (CH_3_-1), 117.61 (C-2), 158.59 (C-3), 101.64 (C-4), 152.64 (C-4a), 164.69 (C-6), 98.47 (C-6a), 164.14 (C-7), 99.17 (C-8), 166.17 (C-9), 55.84 (OCH_3_-9), 103.41 (C-10), 137.79 (C-10a), 108.81 (C-10b). Those data were consistent with literature [10,11,12,13]. Thus, compound **3 **was identified as 3,7-dihydroxy-9-methoxy-1-methyl-6*H*-dibenzo[*b,d*]pyran-6-one, named alternariol 9-methyl ether.

Compound **4 **was isolated as yellow needle-like crystals (MeOH); m.p. 223–237 °C; UV (MeOH), λ_max_ 234 nm; The molecular formula C_16_H_14_O_7_ was assigned by HR-ESI-MS *m/z* 319.0814 [M+H]^+^ (calcd. 319.0813); ^1^H-NMR (DMSO-*d*_6_) δ (ppm): 6.25 (1H, s, OH-1), 3.67 (3H, s, OCH_3_-3), 6.17 (1H, s, H-4), 1.69 (3H, s, CH_3_-4a), 11.35 (1H, s, OH-7), 6.84 (1H, d, *J* = 2.36 Hz, H-8), 3.92 (3H, s, OCH_3_-9), 7.64 (1H, d, *J* = 2.36 Hz, H-10); ^13^C-NMR (DMSO-*d*_6_) δ (ppm): 141.6 (C-1), 172.1 (C-2), 146.8 (C-3), 55.1 (OCH_3_-3), 125.1 (C-4), 68.5 (C-4a), 29.2 (CH_3_-4a), 163.6 (C-6), 101.1 (C-6a), 163.5 (C-7), 102.5 (C-8), 165.9 (C-9), 56.2 (OCH_3_-9), 106.9 (C-10), 135.1 (C-10a), 130.0 (C-10b). After comparing the data with those reported in the literature [14,15], compound **4 **was identified as 1,7-dihydroxy-3,9-dimethoxy-4a-methyl-6*H*-dibenzo[*b,d*]pyran-2,6-(4a*H*)- dione, named botrallin.

### 3.5. Antibacterial Activity Assay

One Gram-positive (*Bacillus subtilis* ATCC 11562), and four Gram-negative (*Agrobacterium tumefaciens* ATCC 11158, *Pseudomonas lachrymans* ATCC 11921, *Ralstonia solanacearum* ATCC 11696 and *Xanthomonas vesicatoria* ATCC 11633) bacteria were selected for the antibacterial activity assay. They were grown in liquid LB medium (yeast extract 5 g/L, peptone 10 g/L, NaCl 5 g/L, pH 7.0) overnight at 28 °C, and the diluted bacterial suspension (10^6^ cfu/mL) was ready for detection. A modified broth dilution-colorimetric assay using the chromogenic reagent 3-(4,5-dimethylthiazol-2-yl)-2,5-diphenyl tetrazolium bromide (MTT) was used to detect the antibacterial activity of the benzopyranones [32]. Briefly, each compound was dissolved in 30% dimethyl sulfoxide (DMSO) at an initial concentration of 2.5 mg/mL. This was diluted with 30% DMSO to obtain a series of concentrations ranging from 0.03125 mg/mL to 2.5 mg/mL. Test sample solutions (10 μL) and prepared bacterial suspensions (90 μL) containing 1 × 10^6^ cfu/mL were added into each well of the 96-well microplate. The negative control well contained 90 μL of the inoculum (1 × 10^6^ cfu/mL) and 10 μL of 30% DMSO. Streptomycin sulfate was used as the positive control. After the plates were agitated to mix the contents of the wells using a plate shaker and incubated in the dark for 24 h at 28 °C, 10 μL of MTT (5 mg/mL in 0.2 mol/L, pH 7.2 phosphate-buffered saline) was added into each well, and the plates were incubated for another 4 h. The microplates incubated with MTT were centrifuged at 1,500 × g for another 20 min. Then the supernatant was aspirated, 200 μL of DMSO was added into each well, and the colored formazan products were extracted for 30 min using a plate shaker. After complete extraction, the plate was centrifuged at 1,500 × g for another 20 min, and then 100 μL of the supernatant (DMSO solution) in each well was transferred to a corresponding well of another 96-well microplate to measure their light absorption values at 510 nm using a microplate spectrophotometer (PowerWave HT, BioTek Instruments, USA). The percentage (%) of bacterial growth inhibition was determined by the formula: 


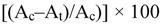


where A_c_ was an average of six replicates of the light absorption at 510 nm of the negative controls, and A_t_ was an average of six replicates of the light absorption at 510 nm of the samples. The median inhibitory concentration (IC_50_) was calculated using the linear relation between the inhibitory probability and concentration logarithm [33].

### 3.6. Antifungal Activity Assay

Rice blast fungus (*Magnaporthe oryzae* P131) was maintained on oatmeal-tomato agar (oatmeal 30 g/L, tomato juice 150 mL/L, and agar 20 g/L) at 25 °C. A spore germination assay was employed to detect the antifungal activity of the compounds [34]. Briefly, the spores were prepared from 7-day-old cultures of *M. oryzae*, according to our previous reports [35,36]. The test compound-acetone solution (25 µL) was mixed with an equivalent volume of fungal spore suspension containing 2 × 10^6^ spores/mL. The mixture was then placed on separate concave glass slides. The final compound concentrations ranged from 3.125 µg/mL to 250 µg/mL in 5% (v/v) acetone. The negative control was 5% acetone, and the positive control was carbendazim with a series of concentrations ranging from 0.78 µg/mL to 50 µg/mL. Three replicates were used for each treatment. The slides containing the spores were incubated in a moist chamber at 25 °C for 7 h. Each slide was then observed under the microscope for observing spore germination status. About 100 spores per replicate were observed to detect spore germination according to the method by Fiori *et al.* [37]. The percentage of spore germination inhibition was determined by the expression: 


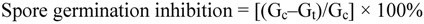


where *G*_c_ is an average of three replicates of germinated spore number in the negative control, and *G*_t_ is an average of three replicates of germinated numbers in the treated sets. The IC_50_ value calculation for the spore germination inhibition was the same as that for antibacterial activity assay.

### 3.7. Antinematodal Activity Assay

The nematode *Caenorhabditis elegans*, which was kindly supplied by Dr. Yuanmei Zuo at the College of Resources and Environmental Sciences, China Agricultural University, was inoculated on the nematode growth medium (NGM) that was cultured previously with *Escherichia coli* OP_50_according to methods of Steiemagle [38]. The NGM plate was full of the worms after 4 to 5 days at 16 °C in darkness. In order to determine the IC_50_ values of the compounds, 5% acetone-water solutions of each sample at 1, 5, 10, 25, 50, 100, and 200 μg/mL were assayed for antinematodal activity. The test nematode dilution (90 μL containing 40–50 nematodes) was added into each well of the sterile 96-well microplate and then, 10 μL of sample stock solution was added into each well and mixed thoroughly. 5% acetone-water solution was used as the negative control. Avemectin B1, which was kindly provided by Dr. Shankui Yuan at the Institute for the Control of Agrochemicals, Chinese Ministry of Agriculture, was used as the positive control with the purity of 97.2%. It was a mixture of avermectin B1a and avermectin B1b in the ratio of 9.5 to 0.5 (w/w). Five replicates were carried out for each treatment, and the experiments were repeated twice. Dead and active nematodes were counted after 24 h. The nematodes were considered to be dead when they did not move by treating with a fine needle as the physical stimuli [39]. The mean percentage of mortality was then calculated. The net percentage of mortality was about 3% by using 5% acetone-water solution as the negative control after 24 h. Nematode recovery was not observed to the dead nematodes. The IC_50_ value calculation for the antinematodal activity was the same as that for antibacterial activity assay.

### 3.8. Acetylcholinesterase Inhibitory Activity Assay

Inhibition of acetylcholinesterase (AChE) by the isolated compounds was investigated using the microplate assay. The assay was based on Ellman’s method [40] using a microplate spectrophotometer (PowerWave HT) with some modifications. In the 96-well plates, 160 μL of pH 7.7 phosphate buffered saline (PBS) solution, 10 μL of 1.0 U/mL enzyme (AChE) solution, 10 μL of DTNB solution, 10 μL of a serially diluted solutions of the isolated compounds, and 9-amino-1,2,3,4-tetrahydroacridine hydrochloride hydrate as the positive control were added. After the mixture was maintained at 30 °C for 15 min, 10 μL of 10 mM ATCI in pH 7.7 PBS solution was added. The absorbance was measured at 405 nm after a period of 30 min incubation. pH 7.7 PBS solution was used as the negative control. The percentage (%) of acetylcholinesterase inhibitory activity was determined using the following expression:


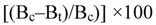


where B_c_ was an average of six replicates of the light absorption at 405 nm of the negative controls, and B_t_ was an average of six replicates of the light absorption at 405 nm of the samples. The IC_50_ value calculation for the acetylcholinesterase activity was the same as that for antibacterial activity assay.

## 4. Conclusions

In this work, four benzopyranones were isolated from the ethyl acetate fraction of the endophytic fungus *Hyalodendriella* sp. Ponipodef12. They were identified as palmariol B (**1**), 4-hydroxymellein (**2**), alternariol 9-methyl ether (**3**), and botrallin (**4**) by means of physicochemical and spectroscopic analysis. The benzopyranones **1**, **2** and **3** were isolated from *Hyalodendriella* sp. Ponipodef12 for the first time. All the isolated benzopyranones showed antibacterial, antifungal, antinematodal and acetylcholinesterase inhibitory activities. To the best of our knowledge, this is the first report on the bioactivity of palmariol B (**1**), as well as the antinematodal and acetylcholinesterase inhibitory activities of the compounds **1**, **2**, and **3**. It is also the first report on the antimicrobial activity of alternariol 9-methyl ether (**3**). The results indicate the potential of the endophytic fungus *Hyalodendriella* sp. Ponipodef12 as a source of active benzopyranones. Some issues such as the mechanisms of action of these benzopyranones, the physiological and ecological roles of the fungus, isolation of the other active compounds including halogen-containing benzopyranones from this fungus, and efficient strategies for increasing benzopyranone content and yield in fermentation culture of *Hyalodendriella* sp. Ponipodef12 need to be further investigated.
